# The Effect of Tattoos on Heart Rate Validity in the Polar Verity Sense Commercial Wearable Device

**DOI:** 10.3390/s25226896

**Published:** 2025-11-12

**Authors:** James W. Navalta, Olivia R. Perez, Rodolfo Mejia, Jennifer A. Bunn

**Affiliations:** 1 Department of Kinesiology and Nutrition Sciences, University of Nevada, Las Vegas, Las Vegas, NV 89154, USA; james.navalta@unlv.edu (J.W.N.); olivia.perez@unlv.edu (O.R.P.); mejiar4@unlv.nevada.edu (R.M.); 2 College of Health Sciences, Sam Houston State University, Huntsville, TX 77340, USA

**Keywords:** photoplethysmography, skin tone, bias

## Abstract

**Highlights:**

**What are the main findings?**

- Heart rate measurements from a commercially wearable device placed over tattoo skin were inaccurate, especially during rest, with error decreasing as exercise intensity increased.
- Skin tone was a significant predictor of measurement error during rest and walking, while tattoo characteristics, such as age and intensity, did not significantly affect heart rate accuracy.

**What are the implications of the main findings?**

- Wearable device manufacturers should consider skin tone and tattoo presence when designing and calibrating sensors, as these factors can introduce bias and reduce measurement accuracy.
- Excluding tattooed individuals from research studies may compromise generalizability and equity, highlighting the need for inclusive methodologies and improved sensor technologies.

**Abstract:**

This study evaluated the accuracy of heart rate (HR) measures of a commercial wearable device on tattooed skin and assessed tattoo characteristics associated with HR accuracy. Participants (*n* = 25) wore a chest strap HR monitor (criterion) and an armband HR monitor (experimental) during rest and self-paced walking and running. Conditions with the experimental device on tattooed and non-tattooed sections of skin were completed, and HR was collected every second and compared via Lin’s correlation (CCC) and the mean absolute percent error (MAPE). Skin tone and tattoo age and intensity were evaluated with HR accuracy. HR from tattooed skin was not accurate during rest (MAPE = 22.9%; CCC = 0.25), walking (MAPE = 7.5%; CCC = 0.68), or running (MAPE = 5.1%; CCC = 0.83). Measures taken on non-tattooed skin were within acceptable standards for accuracy throughout all three conditions (MAPE < 5%; CCC > 0.90). Skin tone was the only characteristic found to contribute to HR accuracy during rest (*p* = 0.046) and walking (*p* = 0.045). No variables loaded for running. The presence of arm tattoos affected HR readings, with the greatest inaccuracy occurring at rest. Specific tattoo characteristics did not statistically contribute to HR accuracy, as shown by the regression analysis. More research is needed to clarify how the varied tattoo characteristics affect HR devices.

## 1. Introduction

Obtaining a tattoo for indigenous peoples was less about artistic practice and more about becoming integrated into the community by providing outward evidence of shared societal values [1,2]. As a result, many tattoo experts were religious leaders who had proven themselves to be worthy of respect [2,3]. Despite the religious and cultural tradition, the practice transformed to carry a stigma in certain countries because tattoos were perceived by people as being associated with criminality and deviant behavior [4,5]. However, the popularity of tattoos tends to fluctuate across time and age demographics [4]. In 2012, it was reported that 38% of 30–39 year olds had a tattoo [6], and this value rose to 46% in the same age group in a 2023 poll [7]. It is presently apparent that the popularity of tattooing has increased, with an estimated 32% of the overall U.S. population having at least one tattoo [7].

Another popular practice is the use of wearable technology, which consists of devices worn on the body and capable of returning metrics such as heart rate, step count, or energy expenditure [8]. From 2016 to the present, wearable technology has been listed among the top world-wide fitness trends [9,10]. With respect to physiological metrics returned from wearable devices, heart rate is generally the most accurate, while energy expenditure is the least, and step count falls in between [8,11]. However, heart rate, particularly when used in an applied setting, may be affected by a number of factors such as device placement, motion artifact, and the influence of ambient light [12,13,14].

One factor that may affect heart rate accuracy returned from wearable devices is the presence of tattoos. Because heart rate in wearable devices are obtained through photoplethysmography-reliant (PPG) technology, the signal may be affected by permanent ink deposited into the skin. While recent guidelines have alluded to the possibility that tattoos may affect the PPG signal of wearable devices [15,16,17,18,19], to our knowledge, no direct investigation has evaluated the effect on heart rate accuracy. Perhaps due to the aforementioned recommendations, some research has begun excluding participants who have tattoos from investigations that utilize wearable devices for obtaining metrics [20,21]. This may be problematic, as excluding individuals from research studies can create bias and compromise the generalizability of the results [22].

To date, no direct investigation has been conducted to determine the effect of tattoos on the accuracy of PPG-based heart rate measurements. Our aim was to systematically determine what effect the presence of a tattoo on the arm would have on heart rate validity measures compared to an electrocardiogram-based criterion measure, as well as a sensor of the same make placed over an area of open skin. We hypothesized, based on the literature, that the sensor placed over a tattooed area would result in less valid measurements, while the sensor placed over the open skin would produce valid heart rate measurements. Additionally, we aimed to determine whether certain tattoo characteristics were associated with heart rate accuracy. We hypothesized that the characteristics of some types of tattoos (color, intensity) would be associated with decreased validity measurements, while other characteristics would not.

## 2. Materials and Methods

### 2.1. Participants

Participants were recruited for this study via convenience sampling. Inclusion criteria were that they had to pass the health screening, have a tattoo on either arm, and have an open skin section on the same arm as the tattoo because of potential interindividual blood flow differences between arms [23]. Participants who were screened and deemed not to require medical clearance to complete exercise according to the American College of Sports Medicine preparticipation health screening recommendations [24] continued with the investigation. Twenty-five adult participants (female *n* = 10, male *n* = 12, gender diverse [transgender MTF, transgender FTM, genderqueer, gender nonconforming, neither exclusively female nor male] *n* = 3) were selected for this study after completing an informed consent form that was approved by the Institutional Review Board (IRB approval #UNLV-2024-528). Gender and ethnicity were obtained using a self-reported questionnaire (Qualtrics, Provo, UT, USA). The number of gender diverse participants were combined (rather than reported individually) to protect confidentiality.

Our previous work using the same devices to evaluate heart rate validity revealed actual power ranging from 0.8034 to 0.9168 with accompanying sample sizes between 5 and 12 participants [25]. To be conservative, we aimed to test a greater number of participants. Demographic characteristics of participants were as follows: age = 33.7 ± 11.4 yrs; height = 170.7 ± 11.9 cm; mass = 82.8 ± 15.6 kg; age of tattoo = 7.7 ± 8.5 yrs. Self-reported race/ethnicity of participants were as follows: Asian *n* = 2, Black or African American *n* = 2, Hispanic, Latino, or Spanish *n* = 6, Multiracial *n* = 1, Native Hawaiian or other Pacific Islander *n* = 2, White *n* = 11, White, some other race, ethnicity, origin *n* = 1.

### 2.2. Protocol

The study consisted of a single day of testing with acute measurements obtained during rest, self-paced walking, and self-paced jogging. Participants were outfitted with a Polar H10 chest strap heart rate monitor (Polar Electro Inc., Kempele, Finland; sampling frequency 1000 Hz), which served as the criterion measure because this family of devices is both valid [26] and reliable [27] at the exercise intensities employed in the current investigation. The experimental devices used in this investigation were the Polar Verity Sense armbands (Polar Electro Inc., Kempele, Finland; sampling frequency 135 Hz). The Polar Verity Sense was chosen because it has shown them to be valid and reliable in a variety of use cases [11,25,28] and it allowed us to place the sensors directly over any tattooed area on the upper or lower arm. All devices (Polar H10, Polar Verity Sense tattoo, Polar Verity Sense open skin) were connected to the PerformTek Data Collector application (Valencell, Inc. Raleigh, NC, USA), which collected the data into a single .csv file per trial and returned heart rate measurements each second.

**Figure 1:**
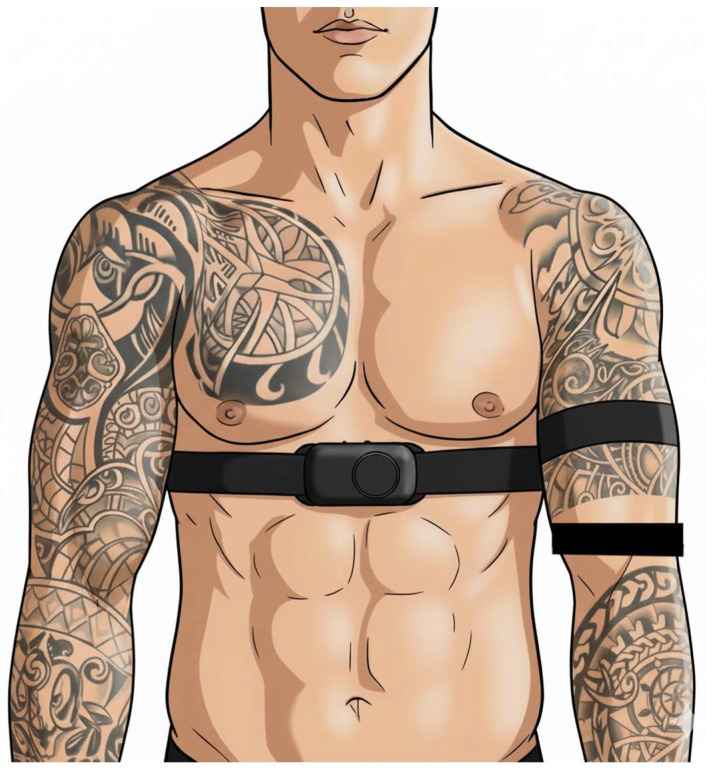
Example schematic of sensor set up. Polar H10 chest strap was placed around the chest. One Polar Verity Sense was placed around the arm over tattooed skin. The other Polar Verity Sense was placed around the same arm on an area of open skin near the sensor over the tattoo.

In this intra-subject design, the criterion device was secured around the chest (see Figure 1). One experimental device was placed over an area of skin at what was perceived by researchers to be the greatest amount of tattoo coverage (see Figure 1). The other experimental device was placed over an area of the skin on the same arm that had no tattoo coverage, as close as possible to the tattooed area to account for potential differences in skin thickness and vascularization (see Figure 1). Once the criterion and experimental devices were secured, the researchers assured all were connected via Bluetooth to the PerformTek Data Collector application, and 5 min of seated rest was completed. Participants then determined their preferred treadmill speeds (WOODWAY 4Front, Waukesha, WI, USA) for walking and running consisting of three self-paced trials per exercise type [29]. During each trial, participants were blinded to the speed displayed on the treadmill and instructed to gradually increase it to a pace they deemed comfortable and sustainable for 5 min of walking or running. The speed was recorded and the procedure repeated three times for walking and three times for running. The speeds from the three trials were averaged to determine individualized self-preferred walking and running speeds. The sample mean speeds were 56.1 ± 18.2 m·min^−1^ for walking and 110.5 ± 25.7 m·min^−1^ for running. Participants completed one 5-min bout of self-paced walking followed by one 5-min bout of self-paced running.

**Figure 2:**
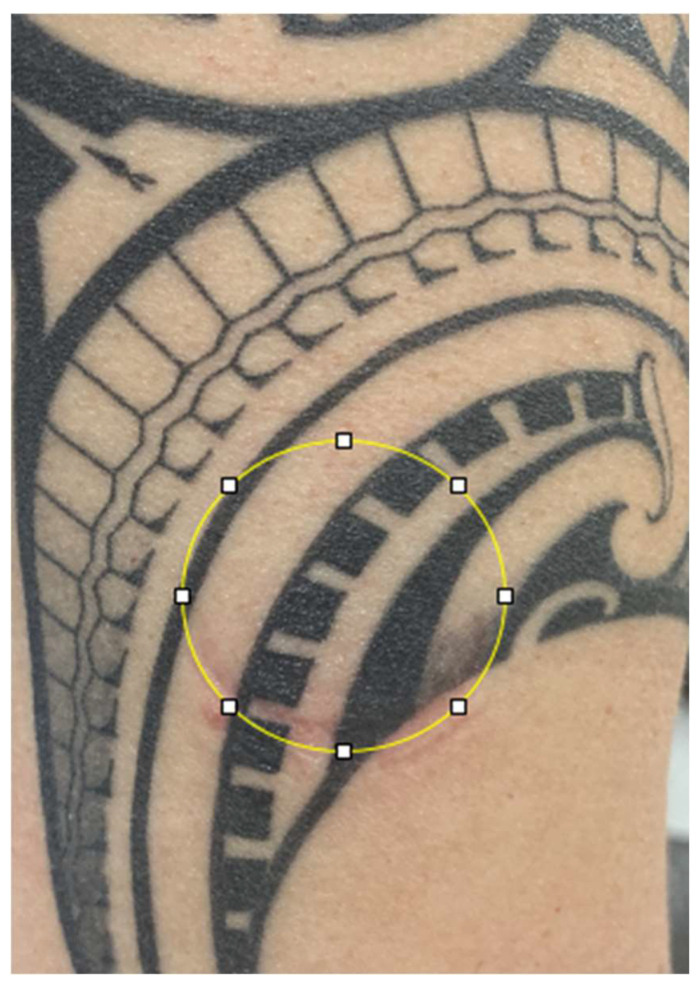
Example of ImageJ procedure on tattooed skin area. Artist attribution: Kiwi Burt.

The validity of the Fitzpatrick Skin Phototype scale has been questioned in individuals with darker skin tone [30] because it does not account for the diversity of skin tones in people of color [31]. The Monk Skin Tone Scale, a 10-shade scale, was used to reduce artificial intelligence bias for users with colored skin [32]. A digital picture of both the tattooed skin area and the non-tattooed skin area was obtained using an Apple iPhone (16e, iOS Version 18.6.2, Cupertino, CA, USA). The images were evaluated using open-source ImageJ software (version 1.54g) [33]. Briefly, the oval selection option was used to click and drag inside the indentation area left by the Polar Verity Sense (see Figure 2), then the mean gray setting was used to obtain values for mean, minimum (min), and maximum (max) intensity scores. Skin tone was determined using the following procedures: the non-tattooed picture was opened in iOS, and the Digital Color Meter was opened concurrently using the hexadecimal view. The Monk Skin Tone Swatches were visually compared to the hexadecimal displayed, opting for the closest swatch.

### 2.3. Data Analysis

Data were downloaded from the PerformTek application and copied into a spreadsheet in Google Sheets (Google, LLC, Mountain View, CA, USA) with calculations for the statistical tests noted below. Error analysis was tested via mean absolute percentage error (MAPE). Linearity was assessed via Pearson’s Product Moment Correlation Coefficient (*r*), and Lin’s Concordance Correlation Coefficient (CCC). Bland–Altman plots were generated with mean bias and 95% confidence intervals and limits of agreement reported. Predetermined thresholds of ≤5% for MAPE [12,34], and ≥0.90 for CCC [12,28] were used. Prediction equations were derived using the Linear Regression function in SPSS (Version 29.0.2.0, IBM Corporation, Armonk, NY, USA) to perform stepwise multiple regression with the predictors of participant mean absolute percentage error being Monk Skin Tone, age of the tattoo, mean intensity of the tattooed area, min intensity of the tattooed area, and max intensity of the tattooed area. The following assumptions were determined: linearity, independence of observations, homoscedasticity, normality of residuals, and multicollinearity. If any of the assumptions were violated, the appropriate action was taken before performing the stepwise multiple regression (i.e., transformation of data, removal of collinear variables).

## 3. Results

**Figure 3:**
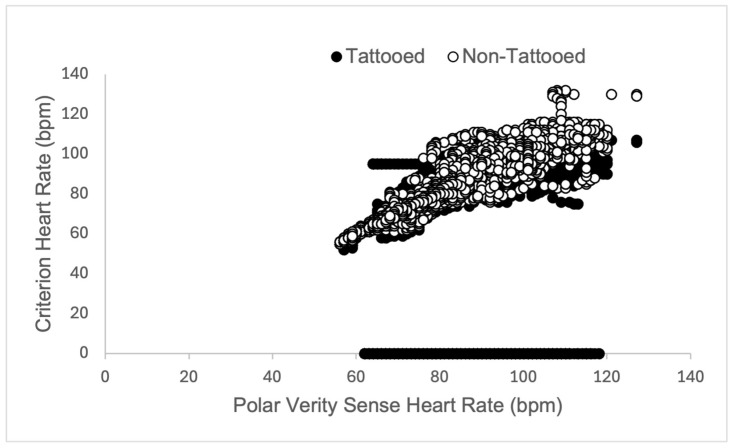
Graphical representation of Pearson Product Moment Correlation heart rate (measured in beats per minute, bpm) returned from the criterion sensor (Polar H10 chest strap) and experimental sensors (Polar Verity Sense) placed over tattooed skin (closed circles) and non-tattooed skin (open circles) on the upper or lower arm during a 5-min rest period in adult participants (*N* = 25).

**Figure 4:**
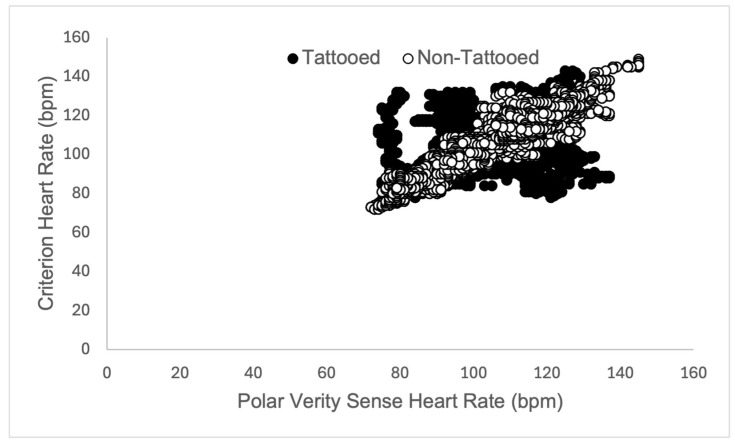
Graphical representation of Pearson Product Moment Correlation heart rate (measured in beats per minute, bpm) returned from the criterion sensor (Polar H10 chest strap) and experimental sensors (Polar Verity Sense) placed over tattooed skin (closed circles) and non-tattooed skin (open circles) on the upper or lower arm during a 5-min self-paced walk in adult participants (*N* = 25).

**Figure 5:**
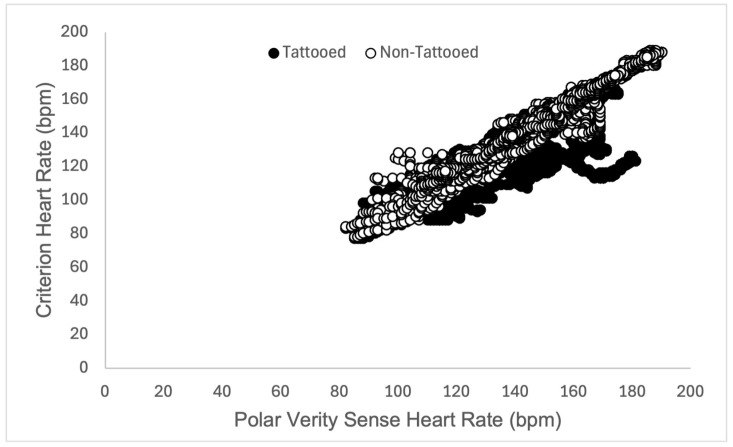
Graphical representation of Pearson Product Moment Correlation heart rate (measured in beats per minute, bpm) returned from the criterion sensor (Polar H10 chest strap) and experimental sensors (Polar Verity Sense) placed over tattooed skin (closed circles) and non-tattooed skin (open circles) on the upper or lower arm during a 5-min self-paced run in adult participants (*N* = 25).

The sensor placed over non-tattooed skin did not function properly in one participant during rest (resulting in 7291 data points rather than 7591, see Table 1). Heart rate validity measures did not meet the threshold for any condition (rest, walk, run) on tattooed skin (see Table 1). Variability in validity measures tended to decrease in tattooed skin as the exercise intensity level increased (see Figure 3, Figure 4 and Figure 5 for a depiction of the Pearson Product Moment Correlation). On the other hand, agreement (in the form of the bias measurement) was narrower during the walk, overestimating heart rate (HR) by nearly 2 beats per minute (bpm), while becoming wider during the run, underestimating HR by 6 bpm. In all cases, the sensor placed over non-tattooed skin met the predetermined validity thresholds (see Table 1).

Closer inspection of the data revealed that in 9 participants, sensor readings were obtained but dropped out to zero at various points during the tattooed skin resting condition. Because of this, the bias in the resting condition was 16.7 bpm lower than the criterion measure. When these 1486 data points were removed, the tattooed skin resting condition did meet validity thresholds (MAPE = 4.2%, CCC = 0.91, *r* = 0.92, R^2^ = 0.84, bias = 0.6 [0.5 to 0.8], and limits of agreement = −11.5 to 12.7).

There was an attempt to determine whether certain tattoo characteristics were associated with heart rate accuracy utilizing individual MAPE percentage over the duration of each trial, and the results of the stepwise multiple regression are shown in Table 2, Table 3 and Table 4. Tattoo characteristics are shown in Table 5. The only significant variable was skin tone in the resting and walking conditions (see Table 2 and Table 3). No significant predictors were noted in the running condition (see Table 4).

## 4. Discussion

The purpose of this study was to utilize an intra-subject design to determine the extent to which tattoos on the arms affected heart rate readings obtained from wearable technology devices. We hypothesized that the sensor placed over a tattooed area would result in less valid measurements, while the sensor placed over the open skin would produce valid heart rate measurements. Additionally, we hypothesized that the characteristics of some types of tattoos (color, intensity) would be associated with decreased validity measurements, while other characteristics would not. Our main findings, using unfiltered second-by-second data, are that resting measurements of the tattooed area appear to be the least valid, followed by the walking condition (did not meet the 5% MAPE or 0.9 Lin’s threshold), and that the self-paced running condition was least affected (barely did not meet MAPE threshold, and not Lin’s). In all conditions, sensors placed over open skin yielded measurements that met all predetermined validity thresholds. Our second hypothesis was incorrect, as we were unable to derive a regression equation that predicted how tattoos would affect heart rate validity during exercise.

The finding that heart rate validity did not meet predetermined thresholds in the resting condition comes with a caveat. We observed dropouts (i.e., zero bpm heart rate readings) in 9 of 25 participants (36%). When these readings were removed from the analysis, the sensor placed over tattooed skin performed similarly to the sensor on non-tattooed skin (meeting the thresholds for all validity measurements) in the resting condition. The only significant predictor of HR validity during rest and walking was skin tone, with darker skin tones associated with worse validity measurements. A similar phenomenon has been described in the literature on pulse oximeters [35,36,37], where darker skin tone is reported to increase the variability of accuracy measurements. Additionally, emerging evidence suggests the technology found in consumer wearable heart rate device sensors may suffer from the same limitations [16]. It is encouraging to note that recently a smartphone adapter was developed, which offers a recalibration to avoid skin tone bias with the aim of overcoming the variation in blood oxygen saturation observed when oximeters are used on darker skin [38]. We suggest a similar effort be applied to consumer grade wearable devices that return a heart rate measurement, accounting for not only skin tone, but also the presence of tattoos. This is important, as health-related research transitions to greater reliance on data provided by consumer grade wearables, and if uncorrected may exacerbate structural health disparities for people with darker skin tones [39], and also for people with tattoos according to the current research findings.

Wearable device manufacturers including Apple, Garmin, and Biovotion, have acknowledged various factors that may affect the heart rate readings, and one factor is the presence of arm tattoos [40]. While many authors have suggested tattoos may affect heart rate measures obtained from wearable devices [15,16,17,18,19], this is the first study to collect experimental data to address the possibility. The Polar Verity Sense was used because we have shown it to have greater accuracy than other wearable devices in a variety of use cases including trail running [25], overground skipping [11], and simulated pickleball [28]. Other authors have reported the Polar Verity Sense to be accurate during high-intensity interval training [41], while performing tactically relevant movements [42], and during laboratory-based and free-living activities [43]. Thus, we supposed that any variation observed in the accuracy was due to the presence and nature of the tattoo. During the exercising conditions, the presence of a tattoo appeared to affect accuracy to a greater degree during the lower intensity activity (walking), than when a higher intensity activity (running) was performed. This phenomenon is counter to what has been reported in studies utilizing non-tattooed individuals, where an increase in intensity results in worsening heart rate measurement error [44,45,46], and appears to be exacerbated in people with darker skin tones [47]. While more research is needed to elucidate the effect of increasing exercise intensity on heart rate accuracy in people with tattoos, we propose the current observations may be due to an increased ability of the sensor to obtain measures through tattooed skin at increased blood flow rates.

This investigation is not without limitations. While every attempt was made to place the sensors over a tattooed area and open skin area in close proximity, this was not possible on every occasion. Future studies should measure and report the absolute and relative positions of each sensor on the arm to determine whether such differences may contribute to the differences observed in the current study. We acknowledge the INTERLIVE recommendations for heart rate-based studies to test at least 45 participants [48]. The current study tested fewer participants, although effect sizes from a previous investigation with the same device suggest a lower number may be appropriate when second-by-second heart rate data are used [25]. Future studies should aim to include an appropriate number of tattooed participants across skin tone categories. Another limitation is the methodology used to determine the skin tones of participants. Photographs were obtained of the participants’ skin were obtained using a cellular phone after exercise, both of which may have impacted the evaluation. Additionally, while the hexadecimal view on the Digital Color Meter allowed for some degree of confidence, a subjective determination using the Monk Skin Tone swatches ultimately had to be made, as the colors did not exactly match what was presented on the scale. Future research using a wider representation of skin tones and more sensitive methodology may be useful as the entire range of the Monk Skin Tone Scale was not represented in the current investigation. As noted previously, future study is needed to determine the mechanism of action for why heart rate accuracy measures appear to improve on tattooed skin as exercise intensity increases.

In conclusion, these findings indicate that the presence of arm tattoos has an effect on heart rate readings obtained from commercial wearable technology device sensors, with the greatest effect observed at rest and variation decreasing as exercise intensity increases. It should be noted that not all tattoos had an impact on heart rate validity measures. In many individual instances, the presence of an arm tattoo did not affect the heart rate validity measurement at all. Thus, care should be taken when excluding participants based on the presence of tattoos. While tattoos affect the validity of heart rate readings obtained via wearable sensors, more research is needed to elucidate the underlying mechanisms, as well as the effect varied characteristics of the tattoo may have (ink color, ink composition, how deep into the epidermis the tattoo is embedded, saturation of the tattooed area, evidence or not of scarring from the tattoo, etc.).

## Figures and Tables

**Table 1:** Validity measures associated with sensors placed over tattooed and non-tattooed skin during rest, walking, and running conditions.

Condition	Data Points	Average (SD)	MAPE (%)	CCC	*r*	R^2^	Bias (95% CI)	Limits of Agreement
Polar H10 Rest	7591	87.3 ± 15.5						
Tattoo Rest	7591	70.6 ± 37.2	22.9	0.25	0.42	0.18	16.7 (15.9 to 17.5)	−49.4 to 82.8
Non-Tattoo Rest	7291	87.4 ± 15.8	2.9	0.96	0.96	0.92	0.02 (0.08 to 0.3)	−8.7 to 9.1
Polar H10 Walk	7557	104.5 ± 15.5						
Tattoo Walk	7557	106.1 ± 14.7	7.5	0.68	0.68	0.47	−1.68 (−2.0 to −1.4)	−25.4 to 22.0
Non-Tattoo Walk	7557	105.1 ± 15.6	2.7	0.97	0.97	0.93	−0.6 (−0.7 to −0.5)	−8.6 to 7.4
Polar H10 Run	7536	140.5 ± 22.7						
Tattoo Run	7536	134.1 ± 23.2	5.1	0.83	0.87	0.74	6.3 (6.0 to 6.6)	−17.4 to 30.0
Non-Tattoo Run	7536	138.9 ± 22.8	2.0	0.98	0.98	0.97	1.6 (1.5 to 1.7)	−6.6 to 9.8

SD = Standard Deviation, CI = Confidence interval, MAPE = Mean absolute percent error, CCC = Lin’s Concordance Correlation Coefficient, *r* = Pearson Product Moment Correlation Coefficient, R^2^ = r-squared. Blacked out areas indicates no data because the Polar H10 is the criterion device.

**Table 2:** Multiple regression model for MAPE (%) in the resting condition.

	*B*	Std. Error	*t*	Sig
Constant	−44.514	58.073	−0.767	0.453
Monk Skin Tone	11.713	5.494	2.132	**0.046**
Age of tattoo	0.926	0.914	1.013	0.324
Tattoo Intensity	0.033	0.481	0.069	0.946
Tattoo Min (intensity)	−0.079	0.414	−0.191	0.851
Tattoo Max (intensity)	0.013	0.276	0.048	0.962

Bold indicates significant predictor variable.

**Table 3:** Multiple regression model for MAPE (%) in the walking condition.

	*B*	Std. Error	*t*	Sig
Constant	−22.729	16.190	−1.404	0.176
Monk Skin Tone	3.281	1.532	2.142	**0.045**
Age of tattoo	−0.340	0.255	−1.336	0.197
Tattoo Intensity	0.150	0.134	1.115	0.279
Tattoo Min (intensity)	0.043	0.115	0.375	0.712
Tattoo Max (intensity)	−0.002	0.077	−0.024	0.981

Bold indicates significant predictor variable.

**Table 4:** Multiple regression model for MAPE (%) in the running condition.

	*B*	Std. Error	*t*	Sig
Constant	0.321	13.123	0.024	0.981
Mink Skin Tone	−0.112	1.241	−0.090	0.929
Age of Tattoo	0.063	0.207	0.303	0.765
Tattoo Intensity	0.043	0.109	0.399	0.694
Tattoo Min (intensity)	0.057	0.093	0.613	0.547
Tattoo Max (intensity)	−0.009	0.062	−0.140	0.890

**Table 5:** Tattoo characteristics associated with participants.

Tattoo Style	*N*	Ink Color(s)	Monk Skin Tone	Tattoo Age (yrs)	Mean Intensity	Minimum Intensity	Maximum Intensity
Fine line	5	Black	4.2 (0.5)	3.3 (3.0)	105.2 (23.1)	35.8 (27.0)	194.6 (49.4)
Illustrative, Realism	3, 4	Black	5.0 (1.6)	6.9 (4.2)	97.7 (10.0)	47.0 (11.6)	208.7 (21.1)
Japanese, Neo Traditional, Traditional	1, 2, 5	Black, blue, brown, green, purple, red	4.8 (1.5)	14.7 (11.6)	109.0 (14.5)	33.6 (15.4)	226.5 (18.8)
Tribal	5	Black	5.4 (0.5)	2.0 (1.7)	106.1 (20.2)	33.8 (7.6)	205.8 (23.5)

## Data Availability

The raw data supporting the conclusions of this article will be made available by the authors on request.

## Abbreviations

The following abbreviations are used in this manuscript:

CCC	Lin’s Concordance Correlation Coefficient
MAPE	Mean Absolute Percentage Error
PPG	Photoplethysmography
*r*	Pearson’s Product-Moment Correlation Coefficient
